# Synthesis of novel oxadiazole derivatives and their cytotoxic activity against various cancer cell lines

**DOI:** 10.55730/1300-0527.3417

**Published:** 2022-03-12

**Authors:** Bilgesu Onur SUCU, Elif Beyza KOÇ

**Affiliations:** 1 Research Institute for Health Sciences and Technologies (SABITA), Center of Drug Discovery and Development, İstanbul Medipol University, İstanbul, Turkey; 2 Department of Medical Services and Techniques, Vocational School of Health Services, İstanbul Medipol University, İstanbul, Turkey

**Keywords:** 1, 2, 4-Oxadiazoles, 1, 3, 4-Oxadiazoles, anticancer activity, glioblastoma cancer cells

## Abstract

Caffeic acid (CA), ferulic acid (FA) and caffeic acid phenethyl ester (CAPE) have a broad anticancer effect on various cell lines. In this study, nine ferulic and caffeic acid-based 1,2,4 and 1,3,4 oxadiazole molecular hybrids were synthesized and their cytotoxic activity was evaluated mainly against Glioblastoma (GBM) cell lines. Compounds 1 and 5 exhibited the highest inhibitory activity against three different GBM cell lines (LN229, T98G, and U87), without toxicity to healthy human mesenchymal stem cells (hMSC). In addition, their cytotoxicity was also evaluated against three additional cancer cell lines and more inhibitory results were found than GBM cell lines. The IC_50_ values of compound 5 in U87, T98G, LN229, SKOV3, MCF7, and A549 cells were determined as 35.1, 34.4, 37.9, 14.2, 30.9, and 18.3 μM. In the light of biological activity studies, the developed compounds have a high potential to lead studies for the development of new drug candidates for the treatment of cancer.

## 1. Introduction

Glioblastoma (GBM) is a very common and aggressive type of primary brain tumor in adults and it was classified as a grade IV glioma tumor by World Health Organization (WHO) [1–3]. Current treatment options such as radiotherapy, chemotherapy, and surgical resection do not increase the survival rate of the patients [4,5]. Temozolomide (3,4-dihydro-3-methyl-4-oxoimidazo-[5,1-d]-1,2,3,5-tetrazine-8-carboxamide, TMZ) has been employed for GBM treatment [6,7]. Since it has a low molecular weight, it could easily pass the blood-brain barrier [8]. In recent years, a combination of TMZ with radiotherapy has been used as standard therapy [9]. However, no further improvement has been noted with TMZ, since some GBM patients are resistant to TMZ treatment [10]. Therefore urgently new therapeutic agents are needed in this area.

**Figure 1:**
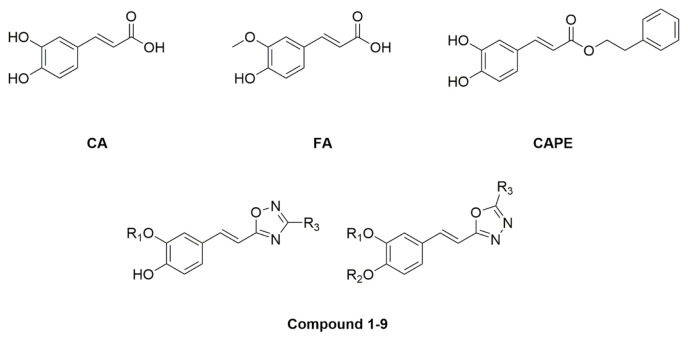
Structures of caffeic acid (CA), ferulic acid (FA), caffeic acid phenethyl ester (CAPE), and Compound 1–9.

Caffeic acid (CA), ferulic acid (FA), and caffeic acid phenethyl ester (CAPE) are biologically active phenolic compounds (Figure 1). While CA and FA are found in many agricultural products such as fruits and vegetables [11–13], CAPE is obtained from propolis in honey bee hives. Their various biological activities have been reported such as antioxidant, antimicrobial, antiinflammatory, and anticancer [14–18]. The phenylpropanoid scaffold of caffeic acid is widely utilized to develop novel biologically active compounds [19]. The oxadiazole core mimicking carboxylic acids, esters and amides can be used as their bioisosters [20]. They also display a wide range of biological and pharmacological activities including antimycobacterial [21,22], analgesic [23], antidepressant [24], COX-2 inhibitors [25], and anticancer [26]. Many of the 1,2,4-oxadiazoles and 1,3,4-oxadiazole compounds are being investigated in biological screening. Due to the potential activities of these compounds, interest towards them is increasing and the acquired data is gaining significance. In our previous study, we synthesized some amides and heterocyclic derivatives and found that the 1,2,4-oxadiazole analog was more potent than the others [27]. Therefore, in this study, we aimed to synthesize some novel hybrid molecules carrying oxadiazoles (Figure 1) and to investigate their anticancer activities against various cancer cell lines.

## 2. Materials and methods

### 2.1. Chemistry

All the chemicals and reagents were purchased from Merck, Sigma-Aldrich, TCI. The purity of the compounds was checked on thin layer chromatography (TLC). Column chromatography purifications were performed on Merck Silica gel 60. Melting points were taken in open capillary tubes using a Stuart SMP30. High resolution mass spectrum (HRMS) was measured using Thermo ORBITRAP Q-EXACTIVE instrument. The 1H and 13C (APT) NMR spectra were measured in CDCl_3_, CD_3_OD, or DMSO-*d*_6_ on a Varian NMR 500 MHz NMR spectrophotometer.

### 2.1.1. General procedure for the synthesis of 3,5-disubstitue-1,2,4-oxadiazole derivatives (1–3)

The mixture of 3,4-Dihydroxyhydrocinnamic acid or ferulic acid (1 mmol), benzamide oxime or 4-(trifluoromethyl) benzamidoxime (1.1 mmol), EDC.HCl (1.2 mmol), and HOBt (1.2 mmol) were stirred in DMF (5 mL). After 1 h, the reaction mixture was heated and stirred overnight at 140 °C. The mixture was cooled to r.t., quenched with LiCl solution and extracted with excess EtOAc. The organic phase dried with Na_2_SO_4_. It was purified by column chromatography with EtOAc:*n*-hexane (1:2) mixture.

### 2.1.2. General procedure for the synthesis of 2,5-disubstitue-1,3,4-oxadiazole derivatives (4–9)

An equimolar mixture of corresponding hydrazide (1 mmol) with caffeic acid/ferulic acid (1 mmol) was refluxed with phosphorus oxychloride (1 mL) for 2–3 h at 100 °C. Reaction mixture was cooled to room temperature and poured into ice cold water. The precipitate obtained was filtered off, washed with water. It was purified by column chromatography with EtOAc:*n*-hexane (1:1) mixture.

#### 2.1.3. Spectral data of the compounds 1–9

##### 2-Methoxy-4-(2-(3-phenyl-1,2,4-oxadiazol-5-yl)vinyl)phenol **(1)**

Light yellow solid. Yield: 68%; mp:166–168 °C. R_f_:0.56 (1:1.5 EtOAc:*n*-hexane). **^1^H NMR (500 MHz, CDCl_3_) δ (ppm)**: 8.15–8.09 (m, 2H), 7.80 (d, *J* = 16.3 Hz, 1H), 7.53–7.45 (m, 3H), 7.15 (dd, *J* = 8.2, 2.0 Hz, 1H), 7.09 (d, *J* = 2.0 Hz, 1H), 6.96 (d, *J* = 8.1 Hz, 1H), 6.90 (d, *J* = 16.3 Hz, 1H), 3.93 (s, 3H). **^13^C NMR **(**APT) (125 MHz, CDCl_3_) δ (ppm):** 175.68, 168.70, 148.37, 147.07, 142.89, 131.23, 128.95, 127.57, 127.12, 127.08, 123.12, 115.05, 109.28, 107.69, 56.06. **HRMS (m/z):** [M–H]^−^ calculated for C_17_H_14_N_2_O_3_:293.0926; found:293.0932.

##### 4-(2-(3-(4-(Trifluoromethyl)phenyl)-1,2,4-oxadiazol-5-yl)vinyl)benzene-1,2-diol **(2)**

Light yellow solid. Yield: 64%; mp:223–225 °C. R_f_:0.48 (1:1 EtOAc:*n-*hexane). ^1^**H NMR (500 MHz, CD_3_OD) δ (ppm):** 8.27 (d, *J* = 8.2 Hz, 2H), 7.84 (d, *J* = 8.2 Hz, 2H), 7.81 (d, *J* = 16.2 Hz, 1H), 7.16 (d, *J* = 2.1 Hz, 1H), 7.07 (dd, *J* = 8.1, 2.1 Hz, 1H), 6.95 (d, *J* = 16.3 Hz, 1H), 6.83 (d, *J* = 8.1 Hz, 1H). **^13^C NMR **(**APT) (125 MHz, CD_3_OD) δ (ppm):** 176.70, 167.29, 148.63, 145.62, 143.88, 132.27, 130.72, 127.55, 126.40, 125.60, 125.02, 122.85, 121.73, 115.21, 113.62, 105.67. **HRMS (m/z):**[M–H]^−^ calculated for C_17_H_11_F_3_N_2_O_3_: 347.0644; found: 347.0649.

##### 2-Methoxy-4-(2-(3-(4-(trifluoromethyl)phenyl)-1,2,4-oxadiazol-5-yl)vinyl)phenol **(3)**

White solid. Yield: 60%; mp:162 °C. R_f_:0.68 (1:1.5 EtOAc:*n-*hexane). **^1^H NMR (500 MHz, CDCl_3_) δ (ppm): **8.24 (d, *J* = 8.1 Hz, 2H), 7.82 (d, *J* = 16.3 Hz, 1H), 7.76 (d, *J* = 8.2 Hz, 2H), 7.17 (dd, *J* = 8.2, 2.0 Hz, 1H), 7.10 (d, *J* = 1.9 Hz, 1H), 6.97 (d, *J* = 8.2 Hz, 1H), 6.90 (d, *J* = 16.3 Hz, 1H), 6.01 (s, 1H), 3.96 (s, 3H). **^13^C NMR (APT) (125 MHz, CDCl_3_) δ (ppm): **176.03, 167.60, 148.40, 146.95, 143.26, 132.66, 130.45, 127.77, 126.89, 125.85, 124.88, 123.15, 122.72, 114.97, 109.18, 107.29, 55.98. **HRMS (m/z):** [M-H]^−^ calculated for C_18_H_13_F_3_N_2_O_3_:361.0800; found: 361.0807.

##### 4-(2-(5-Phenyl-1,3,4-oxadiazol-2-yl)vinyl)benzene-1,2-diol **(4)**

Yellow-brown solid. Yield: 62%; mp: 210 °C decompose, R_f_: 0.68 (2:1 EtOAc:*n-*hexane). **^1^H NMR (500 MHz, DMSO-*d6*) δ (ppm): **8.10–8.07 (m, 2H), 7.63–7.56 (m, 4H), 7.16 (d, *J* = 2.1 Hz, 1H), 7.09 (dd, *J* = 8.2, 2.1 Hz, 1H), 6.99 (d, *J* = 16.3 Hz, 1H), 6.82 (d, *J* = 8.1 Hz, 1H). **^13^C NMR (APT) (125 MHz, DMSO-*d6*) δ (ppm): **164.48, 162.92, 148.01, 145.66, 139.67, 131.86, 129.39, 126.55, 126.25, 123.47, 120.64, 115.91, 114.58, 105.95. **HRMS (m/z):**[M+H]^+^ calculated for C_16_H_12_N_2_O_3_: 281.0926; found: 281.0911, [M+Na]^+^ calculated for C_16_H_12_N_2_O_3_: 303.0746 found: 303.0729.

##### 4-(2-(5-(Furan-2-yl)-1,3,4-oxadiazol-2-yl)vinyl)benzene-1,2-diol **(5)**

Yellow-brown solid. Yield: 62%; mp: 210 °C decompose, R_f_: 0.46 (3:1 EtOAc:*n-*hexane). **^1^H NMR (500 MHz, CD_3_OD) δ (ppm): **7.85 (d, *J* = 1.7 Hz, 1H), 7.55 (d, *J* = 16.3 Hz, 1H), 7.32 (d, *J* = 3.5 Hz, 1H), 7.14–7.11 (m, 1H), 7.01 (dd, *J* = 8.1, 1.7 Hz, 1H), 6.87 (d, *J* = 16.3 Hz, 1H), 6.81 (d, *J* = 8.2 Hz, 1H), 6.73 (dd, *J* = 3.5, 1.8 Hz, 1H). **^13^C NMR (APT) (125 MHz, CD_3_OD) δ (ppm): **165.87, 158.05, 149.59, 147.86, 147.00, 142.04, 140.43, 128.03, 122.56, 116.60, 115.69, 114.67, 113.48, 106.10. **HRMS (m/z):** [M+H]^+^ calculated for C_14_H_10_N_2_O_4_: 271.0719; found: 271.0704.

##### 2-Methoxy-5-(2-(5-phenyl-1,3,4-oxadiazol-2-yl)vinyl)phenol **(6)**

White solid. Yield: 50%; mp:164–166 °C. R_f_: 0.62 (2:1 EtOAc:*n-*hexane). **^1^H NMR (500 MHz, CDCl_3_) δ (ppm): **8.13–8.10 (m, 2H), 7.56–7.50 (m, *J* = 12.7, 6.8, 3.9 Hz, 4H), 7.22 (d, *J* = 2.1 Hz, 1H), 7.08 (dd, *J* = 8.3, 2.1 Hz, 1H), 6.95 (d, *J* = 16.3 Hz, 1H), 6.89 (d, *J* = 8.3 Hz, 1H), 3.94 (s, 3H). **^13^C NMR (APT) (125 MHz, CDCl_3_) δ (ppm): **164.68, 164.00, 148.45, 146.19, 138.88, 131.79, 129.20, 128.65, 127.07, 124.10, 121.15, 112.79, 110.86, 108.30, 56.19. **HRMS (m/z):** [M+H]+ calculated for C_17_H_14_N_2_O_3_: 295.1083; found: 295.1068.

##### 5-(2-(5-(Furan-2-yl)-1,3,4-oxadiazol-2-yl)vinyl)-2-methoxyphenol **(7)**

White solid. Yield: 50%; mp:166–168 °C. R_f_: 0.57 (2:1 EtOAc:*n-*hexane). **^1^H NMR (500 MHz, CDCl_3_) δ (ppm): **7.66 (dd, *J* = 1.7, 0.6 Hz, 1H), 7.53 (d, *J* = 16.4 Hz, 1H), 7.21 (dd, *J* = 3.5, 0.6 Hz, 1H), 7.19 (d, *J* = 2.1 Hz, 1H), 7.08 (dd, *J* = 8.3, 2.1 Hz, 1H), 6.92 (d, *J* = 16.3 Hz, 1H), 6.88 (d, *J* = 8.3 Hz, 1H), 6.62 (dd, *J* = 3.5, 1.8 Hz, 1H), 3.94 (s, 3H). **^13^C NMR (APT) (125 MHz, CDCl_3_) δ (ppm): **164.06, 156.85, 148.49, 146.17, 145.81, 139.74, 139.26, 128.56, 121.23, 114.15, 112.76, 112.36, 110.84, 107.82, 56.20. **HRMS (m/z):** [M+H]+ calculated for C_15_H_12_N_2_O_4_: 285.0875; found: 285.0860.

##### 2-Methoxy-4-(2-(5-phenyl-1,3,4-oxadiazol-2-yl)vinyl)phenol **(8)**

Yellow solid. Yield: 56%; mp:120–124. R_f_: 0.57 (2:1 EtOAc:*n-*hexane). **^1^H NMR (500 MHz, CD_3_OD) δ (ppm): **8.11–8.08 (m, 2H), 7.65 (d, *J* = 16.3 Hz, 1H), 7.61–7.57 (m, 3H), 7.25 (d, *J* = 2.0 Hz, 1H), 7.13 (dd, *J* = 8.2, 2.0 Hz, 1H), 6.94 (d, *J* = 16.3 Hz, 1H), 6.81 (d, *J* = 8.2 Hz, 1H), 3.92 (s, 3H). **^13^C NMR (APT) (125 MHz, CD_3_OD) δ (ppm): **166.75, 165.13, 152.95, 150.17, 142.10, 133.10, 130.38, 130.22, 127.83, 126.82, 124.86, 124.12, 117.12, 111.05, 105.73, 56.37. **HRMS (m/z):** [M+H]+ calculated for C_17_H_14_N_2_O_3_: 295.1083; found: 295.1068.

##### 4-(2-(5-(Furan-2-yl)-1,3,4-oxadiazol-2-yl)vinyl)-2-methoxyphenol **(9)**

Yellow solid. Yield: 52%; mp: 178–180 °C. R_f_: 0.61 (2:1 EtOAc:*n*-hexane). **^1^H NMR (500 MHz, CD_3_OD) δ (ppm): **7.87 (dd, *J* = 1.2 Hz, 1H), 7.62 (d, *J* = 16.3 Hz, 1H), 7.33 (d, *J* = 3.4 Hz, 1H), 7.25 (d, *J* = 1.9 Hz, 1H), 7.13 (dd, *J* = 8.2, 1.9 Hz, 1H), 6.95 (d, *J* = 16.3 Hz, 1H), 6.81 (d, *J* = 8.2 Hz, 1H), 6.75 (dd, *J* = 3.5, 1.8 Hz, 1H), 3.92 (s, 3H). **^13^C NMR (APT) (125 MHz, CD_3_OD) δ (ppm): **166.10, 157.96, 150.22, 147.81, 142.31, 140.50, 126.72, 124.21, 117.14, 115.58, 113.46, 111.04, 105.36, 56.37. **HRMS (m/z):** [M+H]+ calculated for C_15_H_12_N_2_O_4_: 285.0875; found: 285.0859. [M+Na]^+^ calculated for C_15_H_12_N_2_O_4_: 307.0694 found: 307.0678.

### 2.2. Biological methods

LN229-GBM (ATCC, CRL-2611), T98G (ATTC, CRL-1690), U87 (ATCC, HTB-14), MCF7 (ATCC, HTB-22), SKOV3 (ATCC, HTB-77), A549 (ATCC, CCL-185) and Primary Human Mesenchymal Stem (hMSC) (UE7T-13 cells no. RBRC-RCB2161; RIKEN, Japan) cells were available in our laboratory. In vitro experiments were conducted using Gibco brand fetal bovine serum (FBS), high and low glucose Dulbecco’s Modified Eagle Medium (DMEM), Penicillin-Streptomycin, L-Glutamine, and Trypsin/EDTA 0.25%. Cytotoxicity assays were performed using the Promega brand CellTiter-Glo® Luminescent Cell Viability Assay (Cat. no. #G7572) and the Corning 96-black plate (Cat. no. #3603).

#### 2.2.1. Cell culture

LN229, T98G, U87, MCF7, SKOV3, and A549 cell lines were used for cell viability. Cancer cell lines were grown with high glucose Dulbecco’s Modified Eagle (DMEM) containing 10% FBS, 1% Penicillin-Streptomycin, and 1% L-Glutamine at 37 °C in a 5% CO_2_ incubator. The cells were grown on a 10 cm (Corning) petri dish. The effect of the most active molecule on the cancer cell line on the healthy cell line was investigated using hMSC cells. hMSC cells were cultured on a 10 cm petri dish with low glucose DMEM medium containing 10% FBS, 1% Penicillin-Streptomycin, and 1% L-Glutamine at 37 °C in a 5 % CO_2_ incubator. Then, to perform cell viability analysis, the cells were removed from the flask with 0.25% trypsin/EDTA. LN229, T98G, U87, MCF7, SKOV3, A549, and hMSC cells were seeded into 96-well black plate at a density of 6 ×10^3^, 5.5 × 10^3^, 6 × 10^3^, 6.5 × 10^3^, 7.5 × 10^3^, 6.5 × 10^3^, and 6.5 × 10^3^ cells per well, respectively.

#### 2.2.2. Analysis of cell viability

Cells were seeded in 96-well black plates and incubated for 24 h at 37 °C in 5% CO_2_. The culture medium was removed and the cells were treated for 48 h in three different wells for each concentration (1, 10, 25, 50, 100, 250 μM) of CA, CAPE, and novel oxadiazole derivatives. Cell Titer-Glo reagent was added to each well after 48 h of treatment, and samples were analyzed on Spectramax (SpectraMax i3x Multi-Mode Detection Platform). Results were standardized relative to cell controls treated with the highest dose (0.1%) of the compound’s solvent, dimethyl sulfoxide (DMSO) (Santa Cruz). IC_50_ values were determined using GraphPad 8.0.2.

#### 2.2.3. Statistical analysis

Experiments were carried out in three sets, each with its own set of results, which were expressed as mean standard error. All statistical comparisons were made using the Student’s t-test, which claimed equal variance. At *p 0.05 and **p 0.01, the differences were declared statistically significant. The data was expressed as a standard error of the mean (SEM).

## 3. Results and discussion

### 3.1. Chemistry

**Figure 2:**
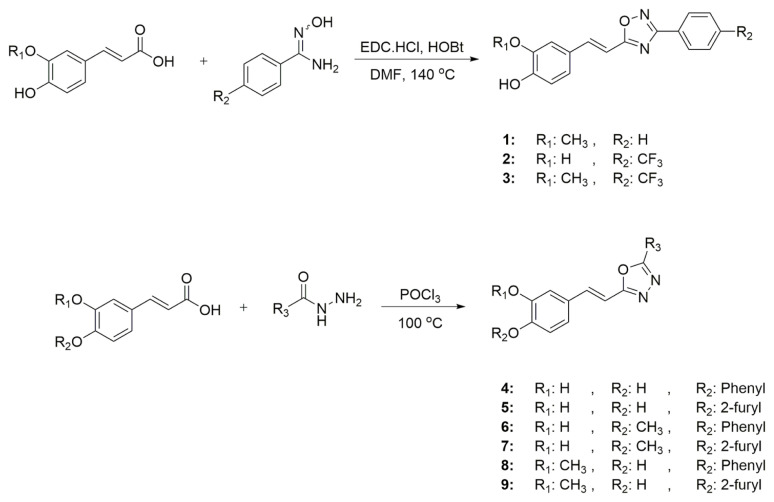
Synthesize of Compound 1–9.

A recent study published by Tripathi et al., showed that the similar hybrid molecules can be effective not only on cancer cells, also used against Alzheimer’s disease (AD) [28]. The inhibition of BACE-1 enzyme with our compound 8 was reported here as a therapeutic approach to treat AD. In our study, the cytotoxic activity of same compound and additional some new oxadiazoles were evaluated as well. In view of this point, some novel 1,2,4- and 1,3,4-oxadiazole analogues (**1**–**9**) were prepared by one-pot reactions (Figure 2). The 3,5-disubstituted-1,2,4-oxadiazoles (**1**–**3**) were obtained by the reaction of caffeic/ferulic acid and the corresponding oxime in the presence of EDC and HOBt. Since we used commercially available trans isomers of caffeic/ferulic acids as starting material, only same isomer was obtained at the end of our method. Compound 1 was synthesized for cytotoxic activity comparison with our previous work [27]. To clarify, the effect of the −CF_3_ group on phenyl moiety, compounds 2 and 3 were prepared and compared their activity with compound 1. The 2,5-disubstituted-1,3,4-oxadiazoles (**4**–**9**) were synthesized to investigate the effect of 1,3,4-oxadiazole ring. These compounds were obtained by the reaction of hydrazide and caffeic/ferulic acid with POCl_3_. The synthesis of the compounds was achieved in 50%–68% yield. Following the synthesis, the crude mixtures were purified by column chromatography and PLC methods using silica gel. All compounds were characterized via ^1^H NMR and ^13^C NMR (APT). Furthermore, calculated and measured m/z values of the compounds were also found compatible in HRMS analysis.

### 3.2. Biological

**Figure 3:**
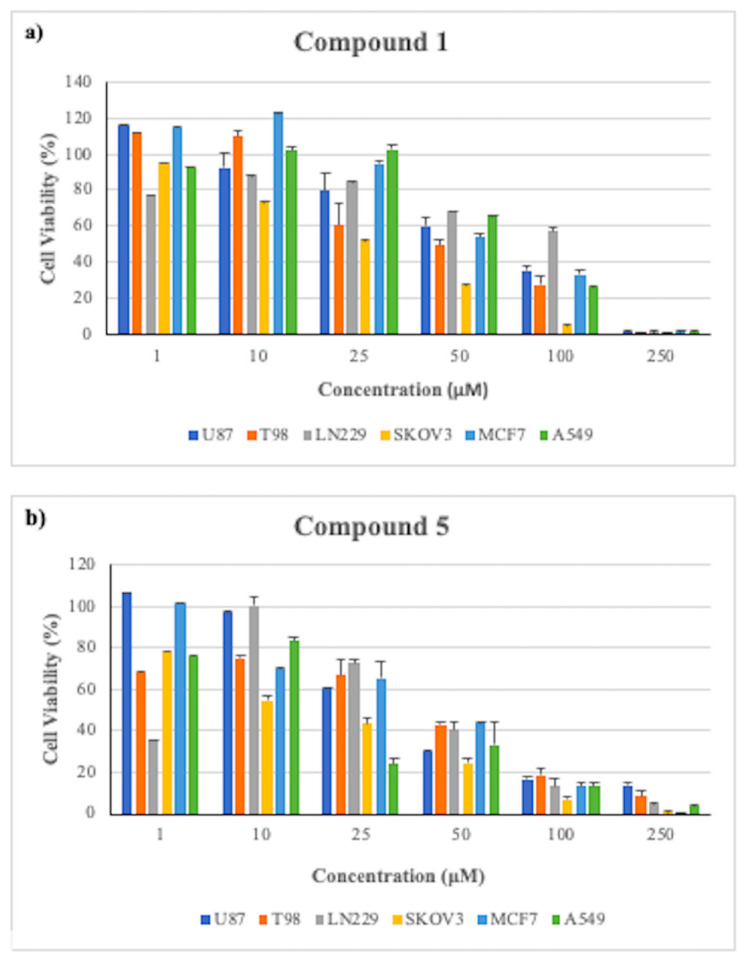
Cytotoxic activity of novel oxadiazole (a) compounds 1 and (b) 5 at 1–250 μM concentrations on different cancer cell lines at 48 h incubation. The error bars show SEM.

Anticancer activity of the synthesized novel oxadiazole derivatives were evaluated against on U87, T98G, and LN229 GBM cell lines. The various concentrations of the derivatives were used to determine IC_50_ values (Table 1) of each compound against the selected cell lines. Among the compounds having 1,2,4-oxadiazole ring, compound 1, exhibited most potent activity on the selected GBM cancer cell lines. On the other hand, among the 1,3,4-oxadiazoles, compound 5 showed the lowest IC_50_ (Figure 3).

IC_50_ values of compound 1 was determined as 60.3, 39.2, and 80.4 μM in U87, T98G, and LN229 cells, respectively. To clarify the effect of −CF_3_ group on the inhibitory activity, the result of compound 3 was compared with compound 1, it was concluded that this group did not increase the activity positively.

In addition, compound 5, possessing 1,3,4-oxadiazole ring was found to have the highest inhibitory activity in comparison to the all other oxadiazoles and reference molecules CA and CAPE. The IC_50_ values of compound 5 in U87, T98G, and LN229 cells were determined as 35.1, 34.4, and 37.9 μM, respectively (Table 1). To examine the effects of furyl and phenyl (R_3_) rings on inhibitory activity, compounds 4 and 5 were prepared and compound 5 with furyl ring showed better inhibitory activity in GBM cell lines. Compounds 6–9 have no significant inhibitory activity on the GBM cell lines. When we compare the inhibitory activity of the compounds 4–5 with compounds 6–9, it was clear that the dihydroxy group of the 1,3,4-oxadiazoles in the phenylpropanoid structure significantly increased the activity.

Cell viability analysis was performed in SKOV3 (ovarian cancer), MCF7 (breast cancer), and A549 (lung cancer) cells for compounds 1 and 5, compound 5 showed higher cytotoxicity than references CA and CAPE in GBM cells lines. The IC_50_ values are listed in Table 2.

In comparasion to GBM cell lines, SKOV3, MCF7, and A549 cells showed higher sensitivity towards compounds 5 and 1. The IC_50_ values of compound 5 were determined as 14.2, 30.9, and 18.3 μM in SKOV3, MCF7, and A549 cells, respectively. Based on these results, compound 5 appears to be more active than both compound 1 and references CAPE and CA in these three different cancer cells.

**Figure 4:**
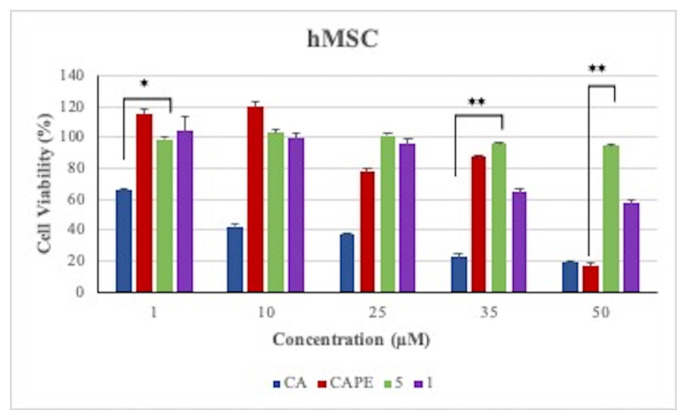
Cell viability results of compounds 1, 5, CAPE, and CA at 1–50 uM concentrations on hMSC cells. The error bars show SEM, *p < 0.05, and **p < 0.01 considered significant (calculated using paired t-test).

In addition, cell viability assay was performed on healthy hMSC in the range of 1–50 μM values to examine the effects of compound 5 and 1 and references CAPE and CA. When the hMSC cells incubated with compounds 5;1 and references CAPE, CA with in the 50 μM (highest concentration) values for 48 h, cell viability was found 94.59%, 57.62% and 16.89%, 19.22%, respectively (Figure 4).

## 4. Conclusion

Novel oxadiazole derivatives were synthesized and evaluated for their anticancer activities in different cancer cells. The most promising results were obtained with compound 1 and 5, carrying 1,2,4-oxadiazole and 1,3,4-oxadiazole moieties, respectively. Compound 5 showed similar activity (35 ± 2 μM) aganist all GBM cell lines. In addition, compounds 1 and 5 significantly inhibit cell proliferation at low concentrations in different cancer cell lines, such as ovarian, breast, and lung. Moreover, the active compounds did not show any toxicity towards the selected nonmalignant cell lines. The results indicate that it is possible to synthesize various derivatives which could be used in further studies to investigate different pathways in cancer.

## Figures and Tables

**Compound 1:**
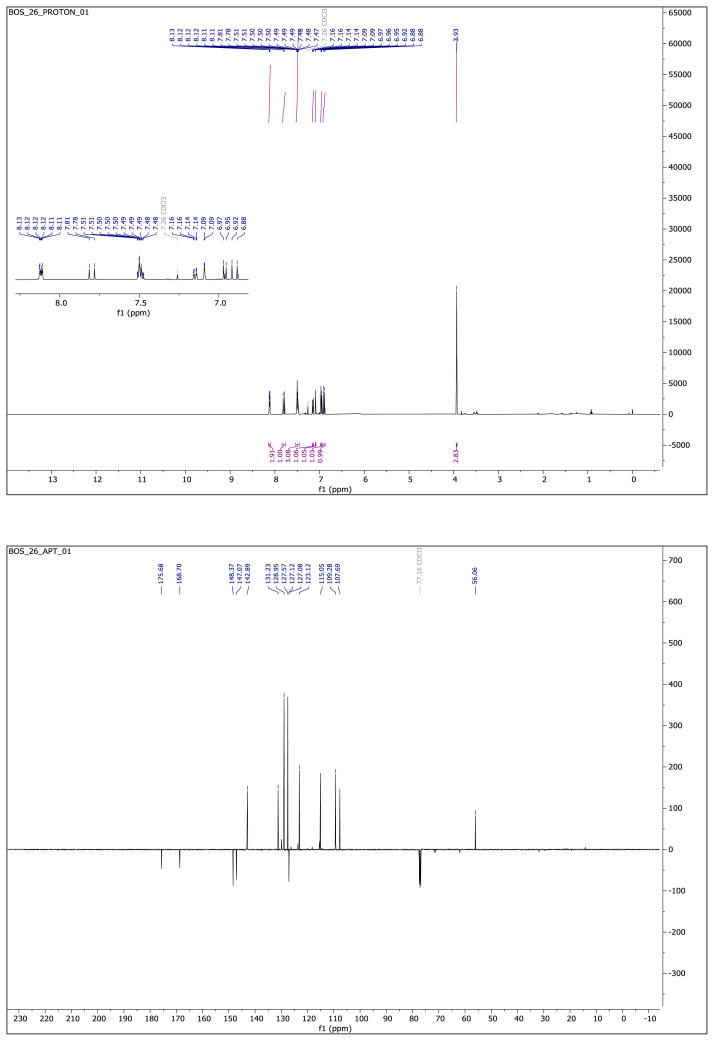


**Compound 2:**
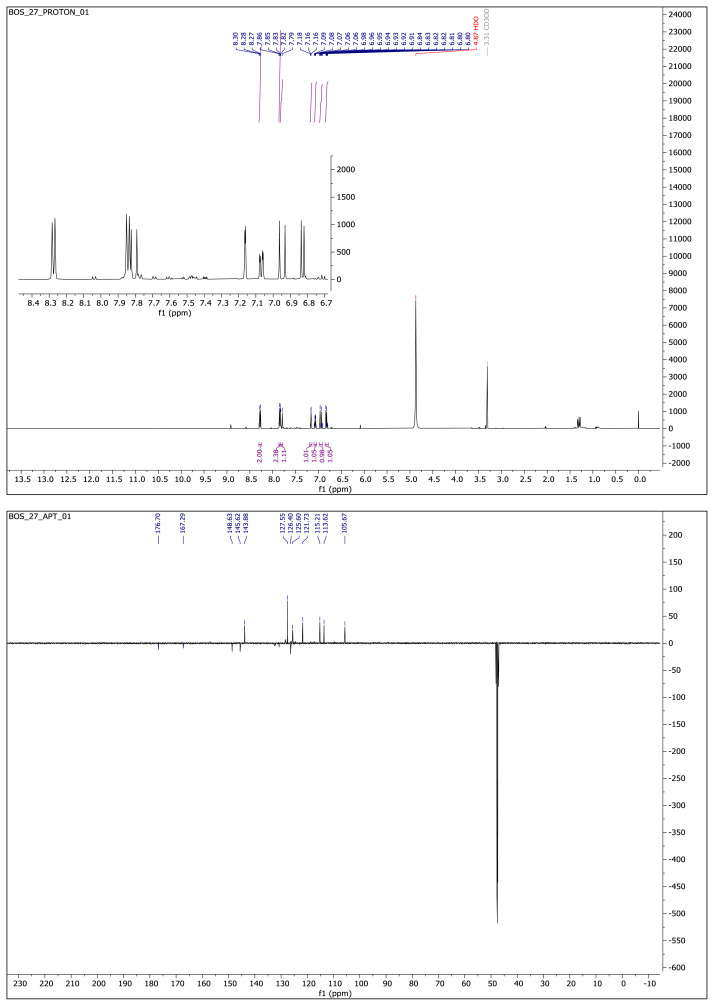


**Compound 3:**
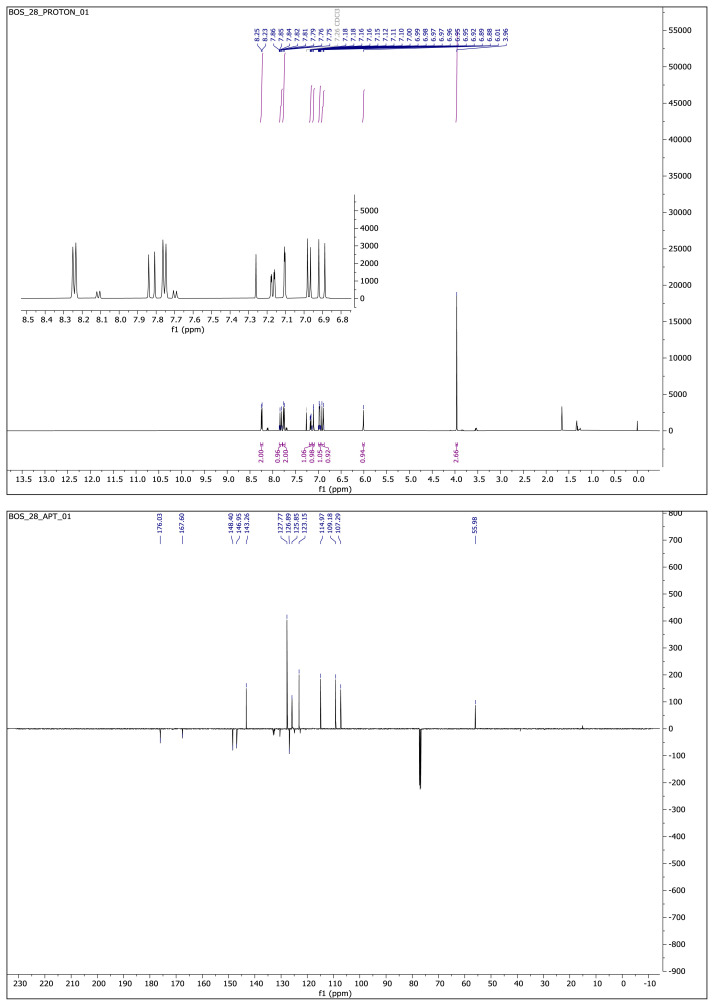


**Compound 4:**
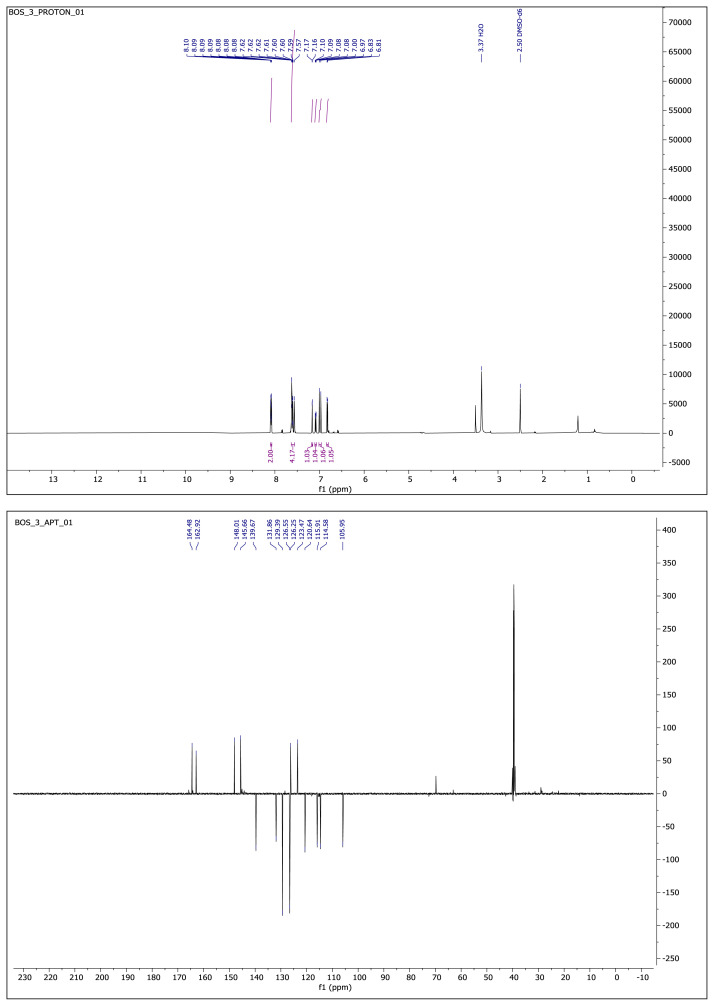


**Compound 5:**
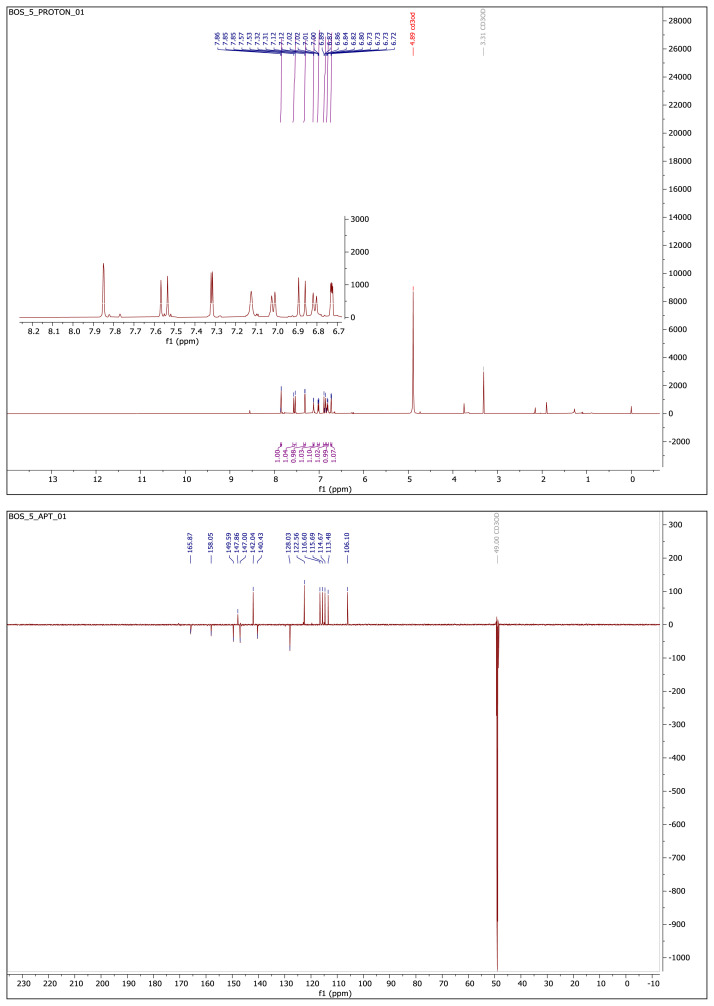


**Compound 6:**
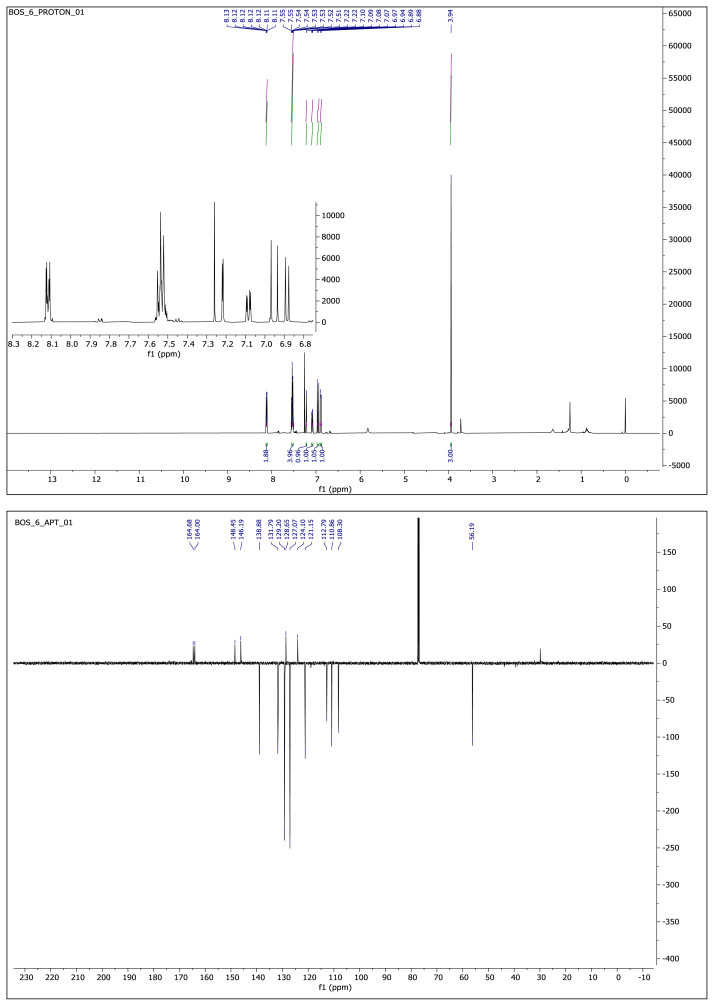


**Compound 7:**
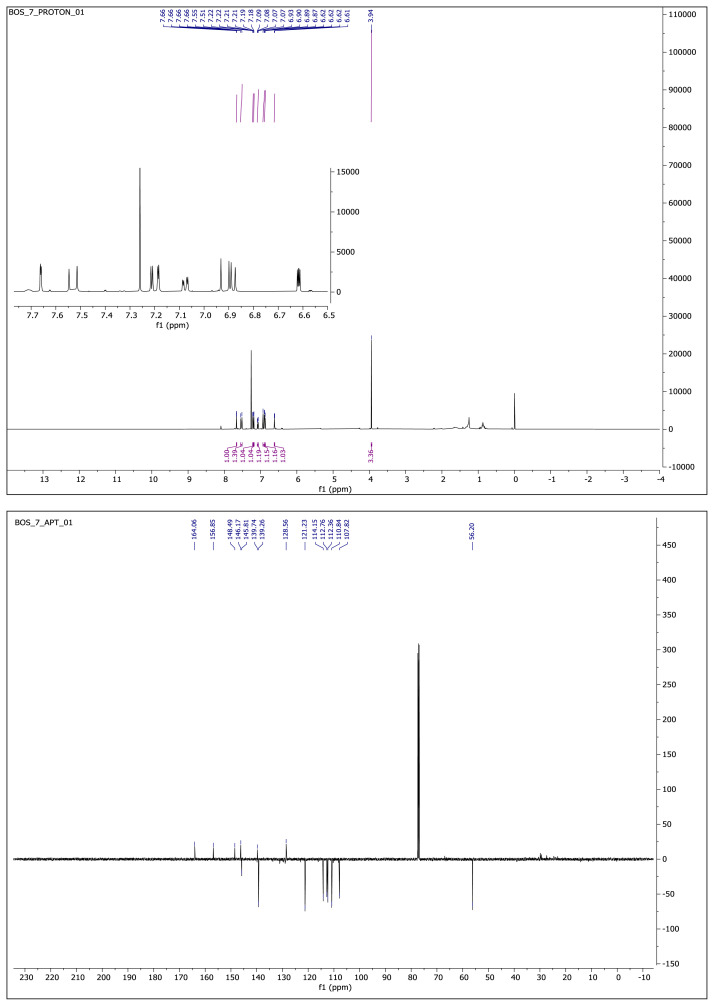


**Compound 8:**
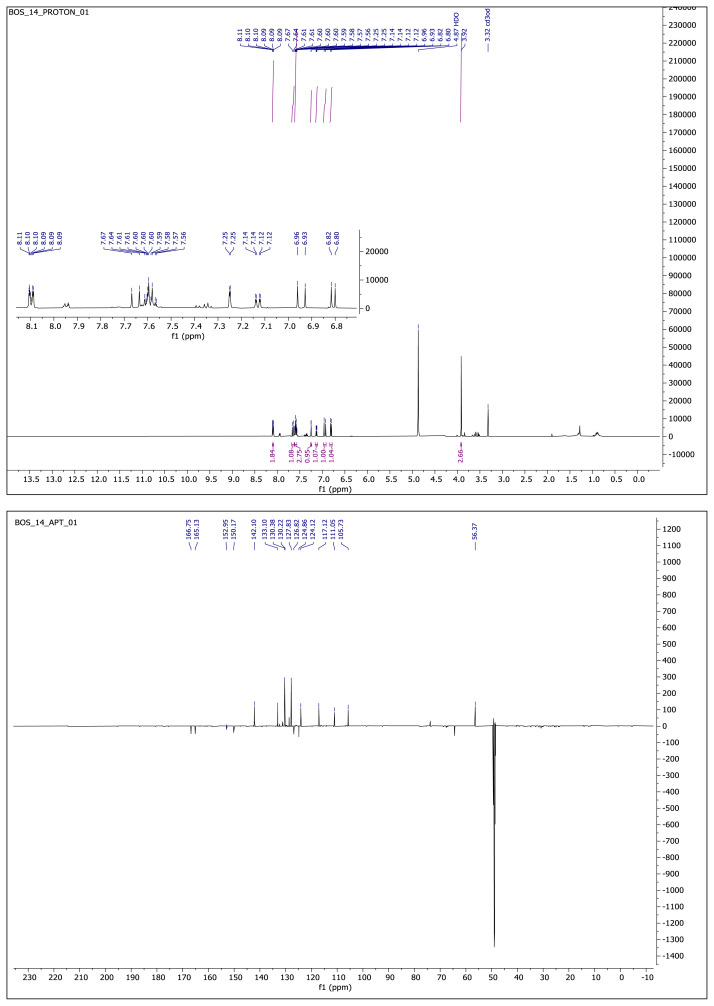


**Compound 9:**
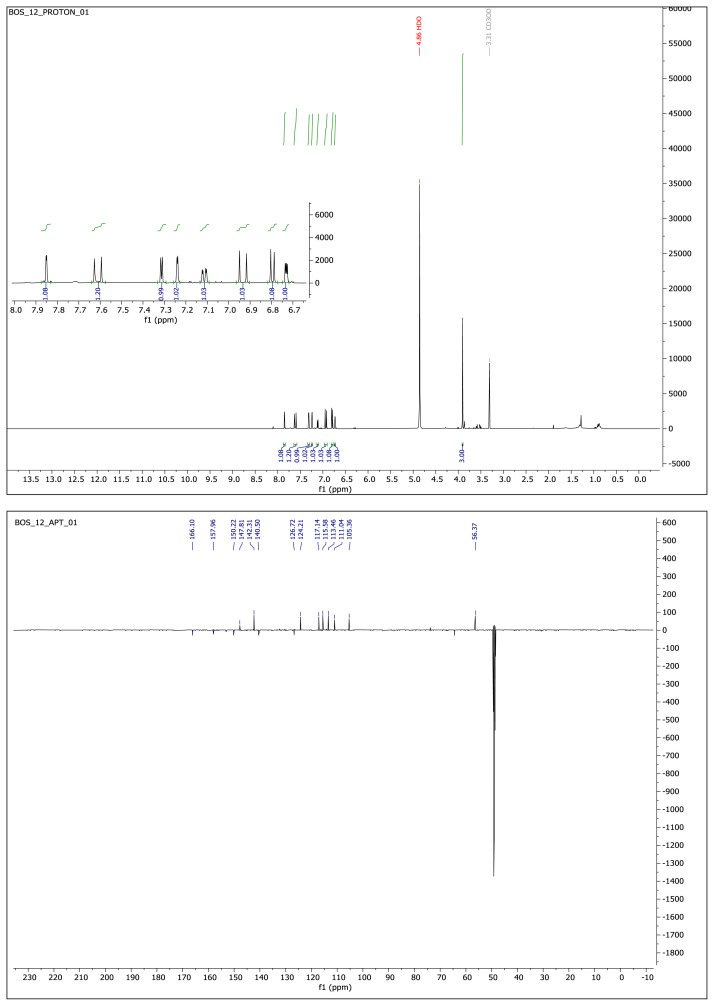


**Compound 1:**
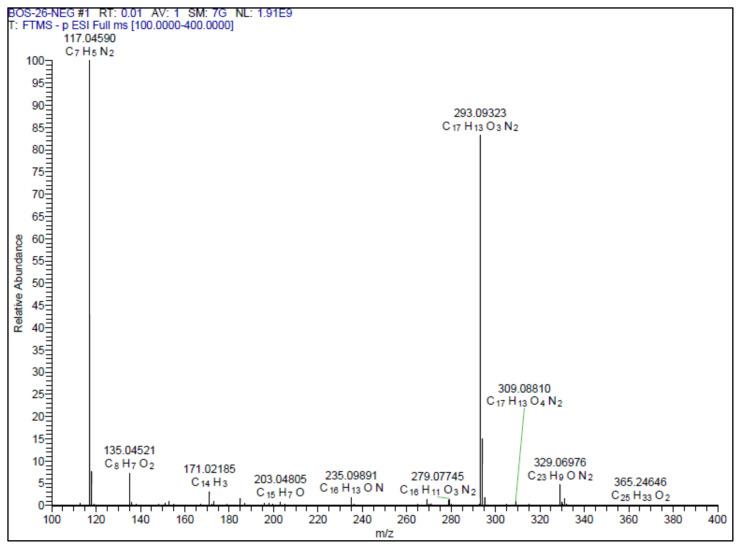


**Compound 2:**
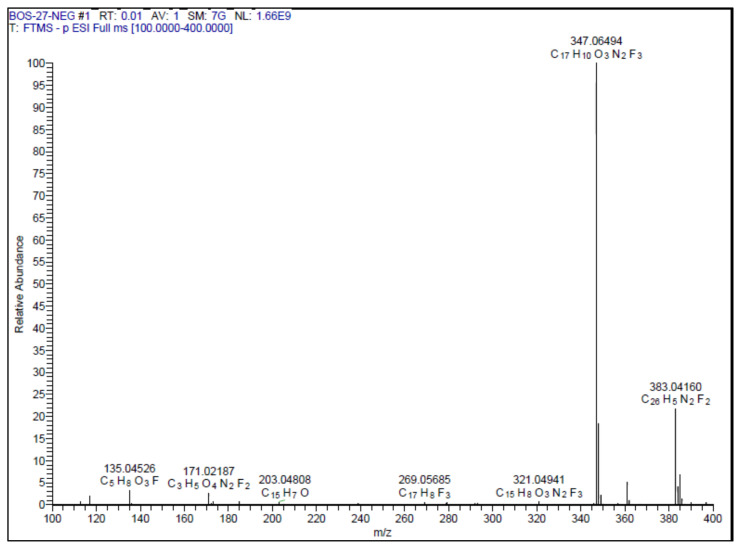


**Compound 3:**
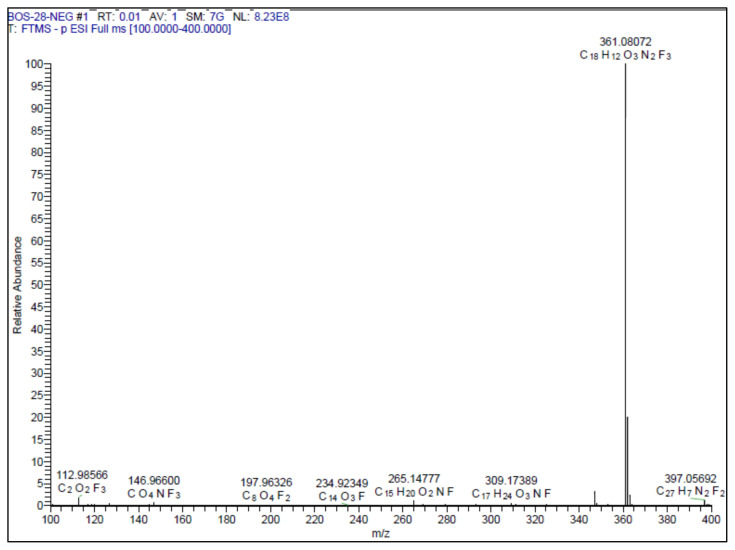


**Compound 4:**
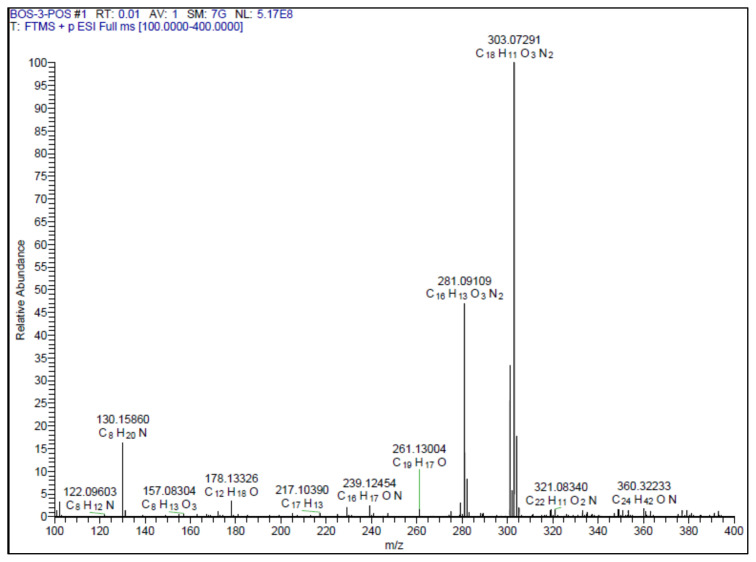


**Compound 5:**
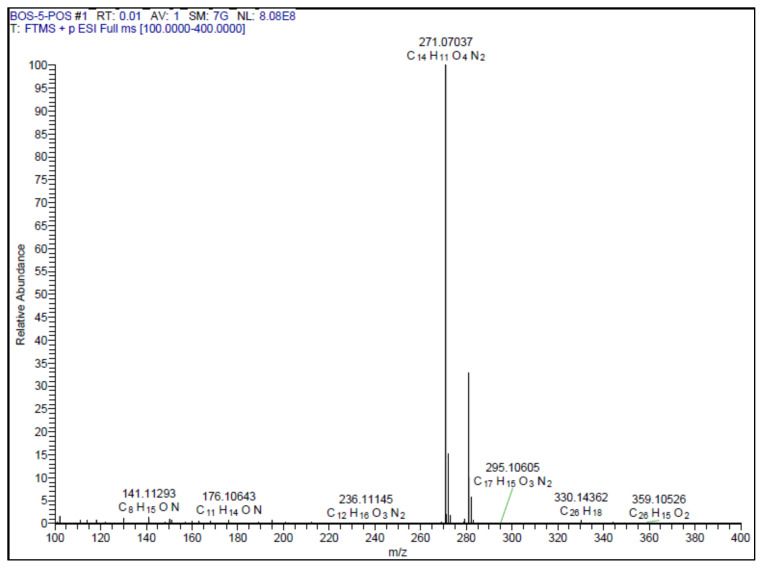


**Compound 6:**
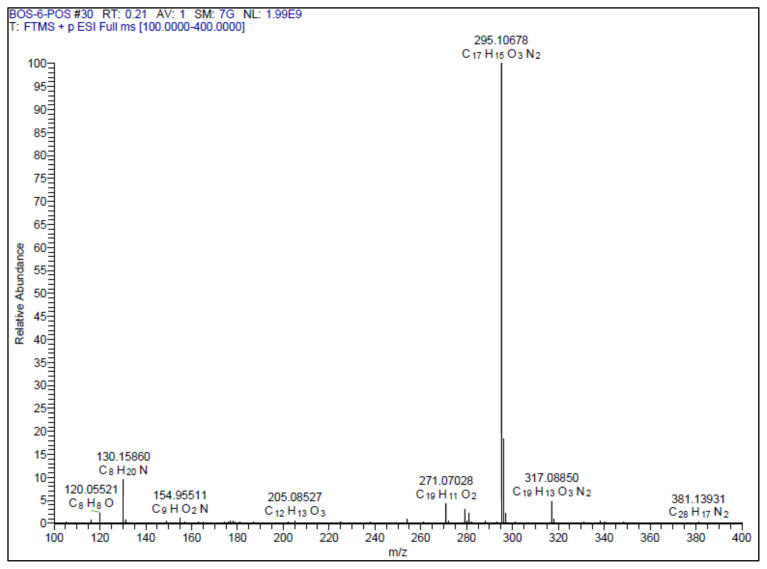


**Compound 7:**
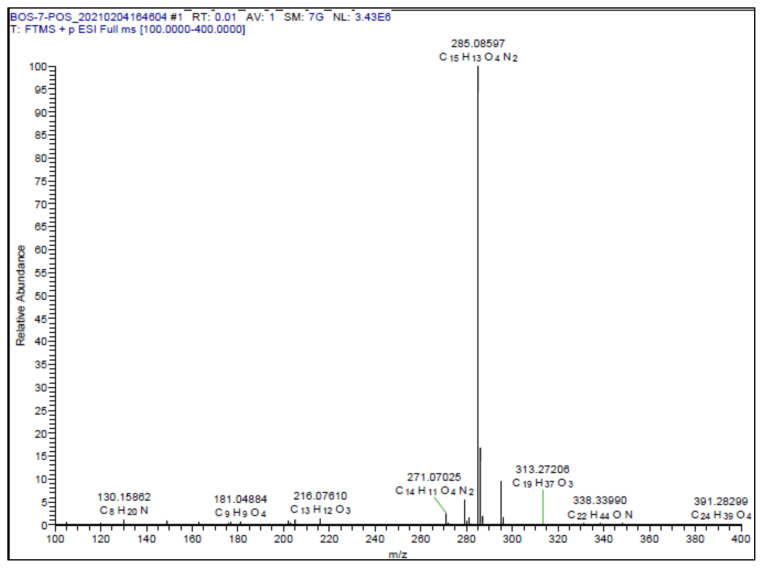


**Compound 8:**
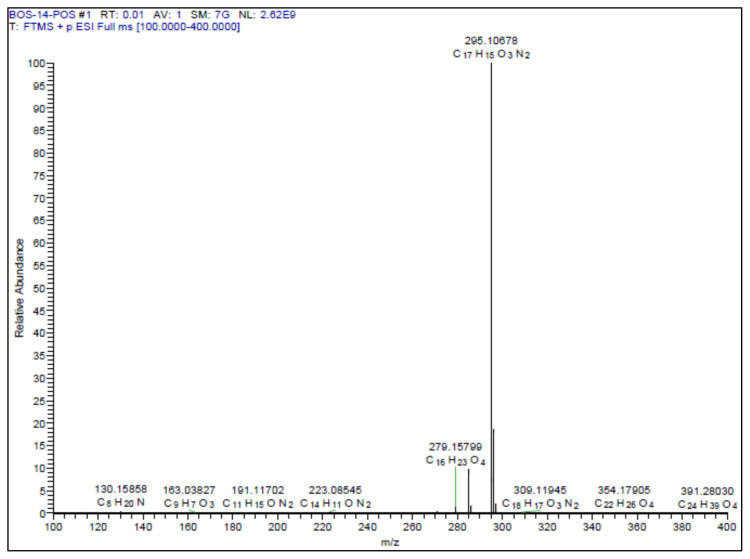


**Compound 9:**
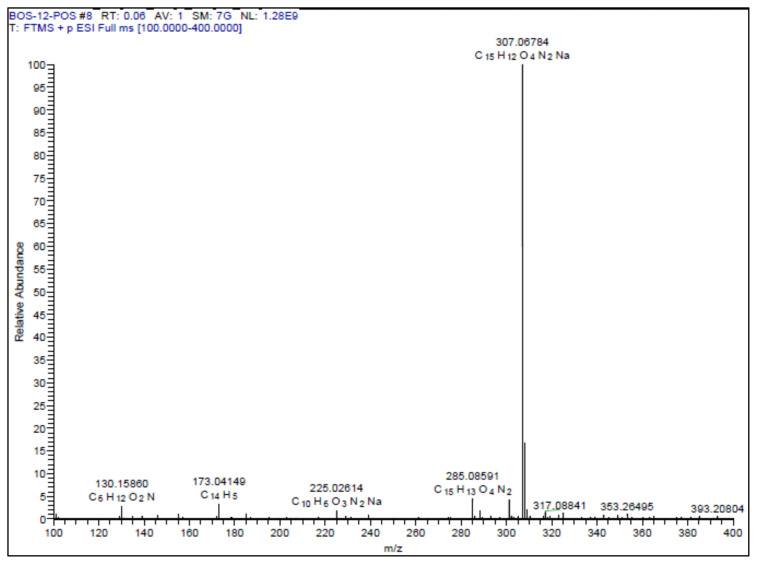


**Table 1:** The IC_50_ values of synthesized novel oxadiazole compounds, CAPE, and CA on U87, T98G, and LN229 cells.

IC_50_ (μM)			
Compound	U87	T98G	LN229
**1**	60.3	39.2	80.4
**2**	117.1	85.5	108.2
**3**	277.1	90.8	51
**4**	95.3	258.6	132.6
**5**	**35.1**	**34.4**	**37.9**
**6–9**	nd*	>250	>250
**CA**	nd*	51.5	56.6
**CAPE**	97.1	97.9	118.2

* nd: not detected

**Table 2:** The IC_50_ values of synthesized novel oxadiazole compounds, CAPE, and CA on SKOV3, MCF7, and A549 cells.

IC_50_ (μM)			
Compound	SKOV3	MCF7	A549
**1**	21.1	70.9	62
**5**	14.2	30.9	18.3
**CA**	38.4	46.1	74.9
**CAPE**	35.5	61.2	191.3
